# Current Trends for Cementation in Prosthodontics: Part 1—The Substrate

**DOI:** 10.3390/polym17050566

**Published:** 2025-02-20

**Authors:** Tatjana Maravic, Claudia Mazzitelli, Eric Mayer-Santos, Edoardo Mancuso, Stefano Gracis, Lorenzo Breschi, Massimo Fuzzi

**Affiliations:** 1 Department of Biomedical and Neuromotor Sciences, University of Bologna, Via San Vitale 59, 40125 Bologna, Italy; claudia.mazzitelli@unibo.it (C.M.); edoardo.mancuso@unibo.it (E.M.); lorenzo.breschi@unibo.it (L.B.); 2 Department of Restorative Dentistry, College of Dentistry, University of São Paulo, São Paulo 14040-904, Brazil; eric.mayer.santos@usp.br; 3 Independent Researcher, 20121 Milano, Italy; sgracis@dentalbrera.com; 4 Independent Researcher, 40126 Bologna, Italy; fuzzi@centromascarella.it

**Keywords:** cementation, luting, substrate, dentin, composite resin, enamel, abutment

## Abstract

With the vast possibilities of restorative dentistry nowadays and the constant development and release of restorative materials with improved mechanical and esthetical properties, there is a growing body of research on adhesive systems and cementation materials, as well as the adequate choices thereof in everyday clinical practice. There are high demands from the dental cements with regard to their adhesion to various substrates and restorative materials, their mechanical properties, resistance to dissolution in the oral environment, esthetic considerations, etc. A material that meets all these requirements is not yet available, and each available material presents certain shortcomings. However, thanks to the advancements in dental material research, polymers-based cements have gained admirable mechanical and esthetic properties, as well as versatility. With the plethora of products available on the market, clinicians are often faced with difficulties in the choice of a material adequate for certain clinical situations. Indeed, important factors to consider are the substrate for cementation and the restoration material. The aim of this review was to provide clear and literature-based clinical recommendations for the adequate cementation of prosthodontic restorations with regard to the cementation substrate. Considering that there is no gold standard protocol applicable in all clinical cases, since the properties of the cementation abutment could substantially differ, important individual considerations must be made for each situation.

## 1. Introduction

In recent years, cementation has been increasingly gaining interest in dentistry, reflecting a growing demand for materials that are chemically compatible with both contemporary materials for prosthodontic restorations and cementation substrates. Among the different factors underlying the clinical success of the cementation process, the substrate is certainly one of the most important ones. Different aspects can have implications in cementation efficacy, such as interactions between the substrate and material, mechanical properties, mechanical and chemical bonding, the condition of the substrate, and combinations of these aspects [1]. The morphology and structure of the dental substrate, including differences between aprismatic enamel, cervical enamel, superficial dentin and deep dentin, can influence the cementation process and quality. Furthermore, caries-infected or-affected dentin, reactive or contaminated dentin, and cut enamel could impair the efficacy of adhesion [2]. It is important to mention that cementation often occurs on non-dental substrates. Prosthetic abutments are often built up with resin composite materials, cast post-and-cores, and sometimes amalgam restorations, making the cementation substrate variable and, at times, rather complex. Therefore, the correct management of the substrate and the correct choice of the cementation material are pivotal for final clinical outcomes, particularly in the case of resin-based cements [1,2,3,4].

Dental luting cements are expected to retain the materials and fill in the gap between the abutment and the restoration, further reinforcing them and improving the longevity of indirect restorations. Thus, in order to be clinically acceptable, they should present adequate resistance to dissolution, high strength under tension, and strong bonding through mechanical interlocking and chemical adhesion, as well as be biologically compatible with the substrate [3,5].

**Figure 1:**
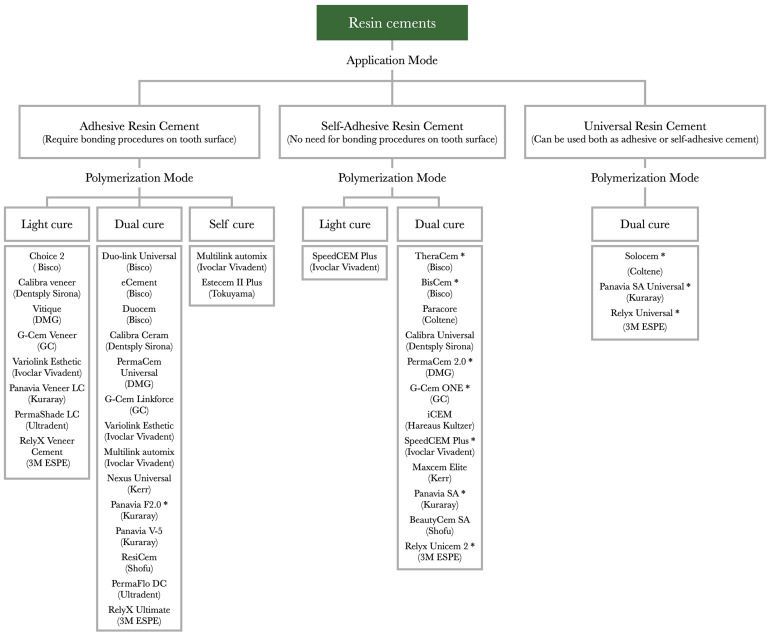
List of resin cements currently available on the market classified by modality of application and polymerization. * Cement contains MDP (10-methacryloyloxydecyl dihydrogen phosphate).

Generally, various materials behave differently depending on the substrate they are placed on. For instance, resin-based cements present an adhesion to dentin that is more susceptible to failure than that to enamel or resin-based restorative materials, and they often have no direct chemical interaction with metal alloys or ceramic materials [6]. On the other hand, glass ionomer cements present chemical interactions with dental substrates and metal alloys, long-term fluoride release, and low coefficients of thermal expansion but lower bond strength to dental substrates and lower mechanical strength when compared with resin-based cements [1,6]. Traditionally, zinc phosphate and glass ionomer cements have been regarded as the most popular materials due to several clinical advantages. However, resin-based cements are currently widely used owing to their easy manipulation, favorable mechanical characteristics and compatibility with a large variety of substrates [4,7]_._ These cements contain resin monomers, inorganic fillers, polymerization initiators and activators. After chemical and/or light-initiated polymerization, an organic polymer network with embedded inorganic fillers with admirable physical and esthetic properties is created. Moreover, several adhesives and resin cements contain 10-methacryloyloxydecyl dihydrogen phosphate (MDP), a monomer that chemically binds to hydroxyapatite and dentin collagen [8,9]. Although the primary bond strength is provided by micromechanical retention, chemical bonds provide additional retention. Resin-based cements can be classified in terms of polymerization and application mode (Figure 1) [10], and the choice of cement is made based on the substrate and clinical situation.

Unveiling the factors involved in the cementation process and the interactions between the large variety of available cementation materials and substrates is crucial for clinicians choosing the most appropriate cementation protocol in order to achieve acceptable longevity for indirect restorations in different clinical situations. However, clinicians are very often faced with clinical situations and substrates they are not sure how to treat and how to cement, particularly when the substrate is “complex” (such as tooth tissue and core build-up material). Thus, the aim of this work was to provide a clinically relevant discussion examining all aspects of cementation substrates and their interactions with different cementation materials. Moreover, clear suggestions for substrate preparation and cement choice will be provided.

## 2. Substrates for Adhesion in Prosthodontics

### 2.1. Enamel

**Figure 2:**
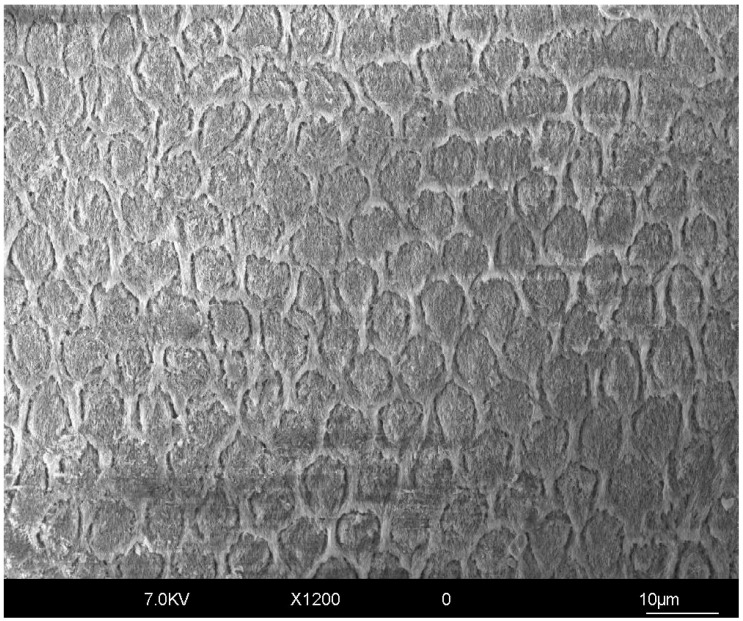
SEM micrograph of enamel surface etched with 37% phosphoric acid gel for 30 s. The prism core material and the interprism areas can be observed. Original magnification of 1200×.

Enamel is a substrate often found after tooth preparation for partial indirect restorations, such as veneers and onlays, as well as, at least in one part of the abutment, after the preparation of teeth for full crowns. To understand the properties of enamel in regard to cementation, it is important to explore its composition and structure. Dental enamel has a unique structure constituting 96% minerals and 4% organic material and water. Its composition provides high hardness, resistance to wear, and stability in physically demanding environments such as the oral cavity [11]. Microstructurally, it consists of nano-sized fibril-like hexagonal hydroxyapatite crystals that are attached in groups (Figure 2). Also, structural blocks of enamel termed prisms and inter-prisms are inserted into that arrangement [11,12]. Generally, human enamel presents different prism orientations among layers. The outer layer of mature enamel is aprismatic [13], while the underlying superficial layer contains parallel prisms named radial enamel. These prisms are orientated radially and intercept the occlusal surface perpendicularly. The inner two-thirds of the enamel present a varying complex of orientation differences referred to as “decussation” [11,12,14]. The decussating groups of enamel prisms, including longitudinally and transversely cut prisms, are termed Hunter–Schreger Bands (HSBs) [14].

Its high mineral content and crystal structure offer enamel important advantages in terms of bond strength. Namely, etching enamel with 35–37% phosphoric acid creates microporosities of enamel that can be efficiently impregnated and interlocked with adhesive resin. It has been undoubtably demonstrated that etch-and-rinse (E&R) adhesive technique provides higher bond strength on enamel compared with the self-etch (SE) technique. Hence, etching is considered a paramount part of any adhesive or cementation procedure on enamel. However, etching alone is often not efficient enough in all clinical situations. For instance, when performing a minimally invasive non-reduction treatment, such as the one for esthetic veneers in the anterior region, the most outer surface of the enamel is aprismatic and can be resistant to acid etching [15], thus requiring a pretreatment of the substrate, such as sandblasting, in order to achieve an acceptable bond strength [4,13,16]. Minimal enamel reduction, or selective reduction, is highly recommended to remove the aprismatic surface, provide optimal bond strength, and allow for a minimal thickness to the restorative material [13,16,17].

The condition and micro-morphology of enamel induce different results [14,18]. In vitro and in vivo studies indicate that the bond strength in cervical regions with few or no HSBs is inferior to that observed in other regions [14,19]. Conversely, regions rich in HSBs present more predictable and successful enamel bonding [20]. Also, the influence of prism orientation on enamel’s tensile properties and bond strength has been reported. Bonding to vertical enamel prisms presents an enamel bond strength twice as strong compared with bonding to parallel ones [18,21,22]. Hence, some studies have recently recommended that cutting the prisms vertically instead of parallelly to their longitudinal axis provides a more favorable marginal configuration for enamel bonding [14,18,21,22,23]. Contemporary techniques for tooth preparation for indirect restorations often take the orientation of prisms into account. For instance, the Morphology-Driven Preparation Technique [23] for partial restorations on posterior teeth involves making a hollow chamfer border on the axial walls so as to increase the surface of adhesion to enamel, ensure the vertical cut of enamel prisms, and improve the esthetic integration of the restoration.

Taking the mechanical and esthetic properties of the current cements and materials for indirect restorations into consideration, resin cements with etch-and-rinse (E&R) or self-etch (SE) used in selective etch mode (SEE) adhesives could be recommended for cementation on enamel. Also, universal resin cements (with the possibility of application with or without a coupled adhesive system) can be applied with adhesives in the E&R mode. Resin cements provide a micromechanical and, in certain cases, chemical retention to dental substrates (Figure 1) [8].

### 2.2. Dentin

The composition and structure of dentin varies in different parts of the tooth due to its tubular structure. Overall, it is composed of approximately 70 weight % or 45 volume % of mineral tissue and 20 weight % or 33 volume % of organic tissue, with water as the remaining fraction [24,25].

**Figure 3:**
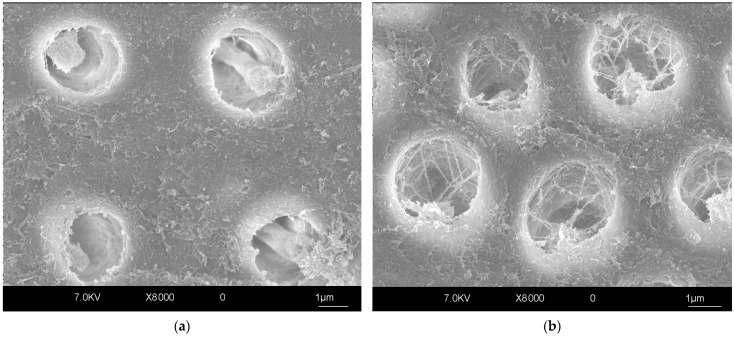
SEM micrograph of dentin surface etched with 37% phosphoric acid gel for 15 s. Original magnification of 12,000×. (**a**) Superficial dentin with wider peritubular areas and fewer dentinal tubules. (**b**) Deep dentin with a higher number of dentin tubules and reduced areas of peritubular dentin.

The presence of tubules is a distinctive characteristic of dentin. This peculiar arrangement of dentinal tissue influences its mechanical behavior, permeability and bonding properties [24,26]. Since dentinal tubules are in a radial disposition and of a conical shape with the wider part facing the pulp, the deeper the dentin, the larger the surface occupied by dentinal tubules in coronal dentin (Figure 3). Hence, superficial dentin presents a higher surface of intertubular dentin, which is mainly an organic matrix constituting collagen fibrils, unlike peritubular dentin, which is high in mineral content. Both intertubular dentin and the tubules are equally important for adhesion to dentin due to interdiffusion with adhesive resins [27,28,29]. Therefore, regional variations in dentin structure could influence the quality of adhesion. Furthermore, the surface water content is higher in deeper dentin, leading to a lower bond strength [30]. Different factors, such as aging, carious and reparatory processes, the preparation of dentin, proximity to pulp tissue, and the use of diverse chemical cleaning or whitening agents, can significantly influence bonding and cementation to dentin [26]. These factors will be elaborated on in the following paragraphs.

Root dentin, on the other hand, has certain features that could affect adhesive bonding and should therefore be discussed. Narrow and deep root canals prevent light from penetrating, which consequently affects the polymerization of resin adhesives and cements [31]. Therefore, if adhesive cementation is required, dual-cure resin-based cements are recommended (Table 1). The degree of conversion would not be adequate with entirely light-polymerizable cements even in the case of glass-fiber post cementation, since an adequate amount of light cannot reach the more apical portions of the root. Self-adhesive resin cements have been demonstrated to perform equally well in fiber post cementation and in more complex adhesive resin cements [32]. Furthermore, the geometry of root dentin is favorable for the development of high shrinkage stresses [31,33], which can lead to debonding and can only be compensated for by the viscoelastic and rheological properties of the luting materials [34].

**Figure 4:**
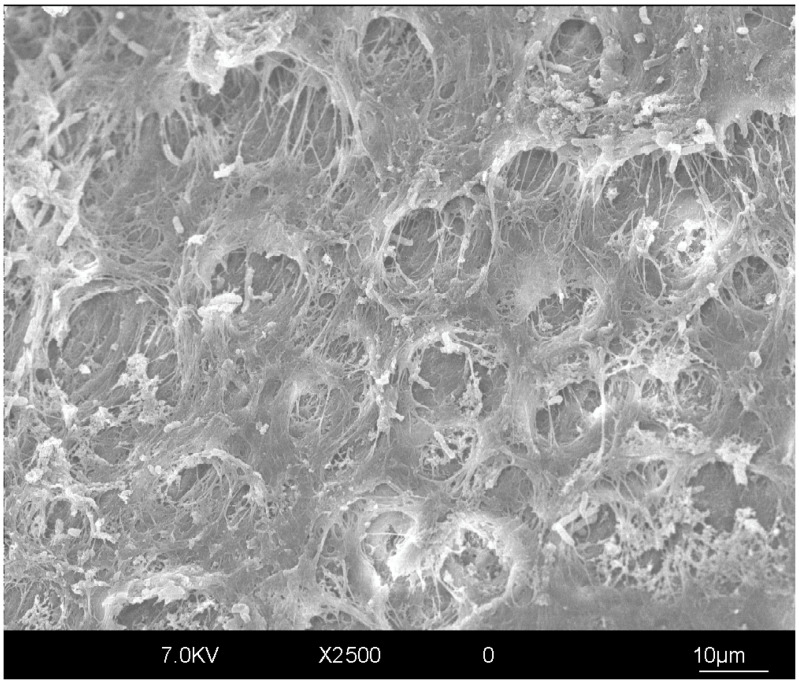
Representative SEM image of primary carious dentin. High degree of dentin demineralization with exposed collagen fibers and bacteria-infected dentin can be noted. Original magnification of 2500×.

Given that in clinical settings, the dentinal substrates available for bonding are rarely completely sound, it is important to emphasize the differences in bonding quality in these cases. Depending on the clinical situation, caries removal method, and extent of the removal of carious dentin (often governed by preferences and personal experience of the operator), the dentinal substrate available for bonding could be partially caries-infected and/or -affected [35]. In fact, an expert statement has recently indicated that interventions in the caries process (if any) should preferably be minimal, with selective carious tissue removal so as to preserve pulp health and with restoration to obtain function and esthetics while interrupting bacterial activity [36]. However, the caries process causes structural changes in dentin (Figure 4), leading to reduced mechanical properties and inevitably having implications in adhesive strength. The mineral content and crystallinity of hydroxyapatite are reduced and are followed by changes in the structure of collagen [37]. This tissue contains a higher percentage of water [38] and is poorly infiltrated by adhesive resins. Consequently, hybrid layers in caries-infected and -affected dentin have been reported to be thicker, poorly infiltrated by bonding agents, and more prone to hydrolytic degradation [39]. The polymerization of dental adhesives on caries-affected dentin was also shown to be less efficient compared with sound dentin [40]. The majority of studies investigating bonding to dentin in vitro have been performed on sound dentin, which might be less relevant form a clinical standpoint, since an operator is most often faced with cavities that include an array of different substrates from enamel to sound dentin, caries-infected or -affected dentin, and sclerotic dentin. The available in vitro research on bonding to caries-affected dentin has shown a 20–50% lower bond strength of this substrate compared with sound dentin [35,41,42]. Furthermore, the immediate bond strength in caries-affected dentin seems to be higher when E&R adhesives are used compared with self-etch (SE) systems [43,44]. However, these differences seem to disappear after short-term aging [44]. Long-term aging studies are necessary to further investigate this matter. On the other hand, long-term clinical retrospective studies have demonstrated a high level of success for posterior composite restorations over time [45,46], even though the majority of posterior cavities contain a caries-affected dentin portion. This disparity could be due to the fact that, as previously mentioned, cavities also contain enamel and sound dentin, superior bonding substrates that are usually the structures directly exposed to the oral cavity, with caries-infected/-affected dentin being in the deeper portions of the cavity. Altogether, this could enhance the clinical durability of restorations.

Sclerotic dentin is another specific dentinal substrate to be considered in terms of adhesive bonding. This dentin is characterized by a superficial layer of hypermineralized dentin, often containing trapped microorganisms, under which is a layer of dentin with denatured collagen fibrils. The tubules are filled with mineral crystallites [28]. It has been shown that resin tags and intertubular dentin hybridization contribute about 20% each to bond strength [27,28,47]. Due to the specific structure of sclerotic dentin, this hybridization is significantly reduced, which could in turn underlie the 25–50% lower bond strength measured on this substrate compared with sound dentin [48,49]. It is plausible to conclude that the standardized etching procedures are inadequate in this case and should be modified. However, a mere prolongation of etching time showed an improvement in some but not in all tested adhesives [50], indicating that this solution might not be sufficient to resolve issues such as bacterial infiltration or denatured collagen fibrils. Pretreatment with EDTA [51] and sandblasting [52] were demonstrated to increase bond strength to sclerotic dentin.

Hence, it is clear that dentin is a highly variable dynamic tissue, and it is therefore difficult to control adhesive bonding and cementation to dentin and to predict the longevity of hybrid layers in each individual case.

Adhesive resin cementation on dentin could be recommended for a variety of clinical cases. The depth of dentin and other aforementioned dentin properties should be taken into consideration. Resin cements combined with E&R or SE adhesives, self-adhesives, or universal resin cements could be used in sound dentin. Self-adhesive cements have demonstrated a similar bond strength in dentin compared to resin cements that require an adhesive in simplified and less technique-sensitive clinical procedures for full crown cementation [53,54]. However, for the cementation of partial restorations that completely rely on the adhesive properties of cements, it is still recommended to use adhesive resin cements [55]. In deeper portions, SE adhesives and cements could be recommended due to lower post-operative sensitivity [56]. For root dentin, dual-curing resin cements or conventional cements, such as glass ionomer or zinc phosphate cements, could be recommended (Table 1). Regardless of the bonding strategy or resin cement used, the materials should always be employed strictly following the manufacturers’ instructions and within the indicated shelf-life [57].

Glass ionomer cements (GICs) should also be taken into consideration for cementing indirect restorations on dentin substrate. GICs present physicochemical bonding to tooth tissue through an ionic interaction with the mineral phase between calcium and the carboxyl ions of hydroxyapatite. This bonding occurs even in the presence of a smear layer, but conditioning improves bond strength. Conditioning produces micro-porosities on the surface of teeth, which increases the area for chemical bonding or micro-mechanical bonding through polymer penetration. Due to the antimicrobial properties of the fluorides released by a GIC, this cement could be recommended in caries-affected dentin for full crown cementation or for the cementation of a post in a root canal. However, GICs present a low mechanical strength, which can compromise the cementation in areas subjected to high stresses [1,4,58,59]. Resin-modified glass ionomer cements (RMGICs) have shown a comparable bond strength to dentin and fluoride release as conventional GICs, with better mechanical properties [60,61].

Another cementation material, traditionally widely used for many years, is zinc phosphate. This material has retentive properties and a high fatigue strength, and it shows a minimal film thickness after cementation (<25 µm) [62]. Retention to an abutment is only promoted by a friction grip, without chemical bonding to enamel or dentin. Also, this tensofrictional retention is facilitated by the mechanical properties of zinc phosphate [63]. Hence, zinc phosphate cement could be considered a choice for the cementation of full crowns. However, its lack of adhesion to substrates, higher solubility, and lower retention compared with other cements have shifted the focus off this material [4,62,64].

### 2.3. Differences Between Enamel and Dentin with Regard to Adhesion

Differences between the dental tissues as substrates for cementation are pronounced in the case of resinous adhesives and cements. Adhesion to dental tissue occurs differently depending on the type of substrate. In the case of E&R materials, it starts with an acid-etching step, which promotes surface demineralization in enamel or dentin. In enamel, the highly mineralized structure formed by the prisms is selectively removed, producing a favorable surface for resin infiltration and micro-retention. The penetration occurs by capillary attraction, even in common hydrophobic agents, since enamel can be extensively dried before bonding. Hence, the micro-mechanical interlocking of the acid-etched surface with the resin tags results in a stable enamel–resin bonding, which is considered the best achievable bond to a dental substrate. This provides a long-term effective seal on restoration margins and protects the underlying tissue from degradation [63,65].

On the other hand, adhesion to dentin substrates is more difficult and less predictable, as dentin presents a heterogeneous morphology and more complex composition than enamel. Acid-etching in dentin substrate exposes collagen fibrils and activates the bound matrix metalloproteinases (MMPs) and cysteine cathepsins (CTs) [66,67,68,69]. These proteases have the potential to degrade the exposed collagen fibrils, decreasing the stability of resin–dentin bonds [70,71,72]. Also, dentin is connected with the pulp tissue via multiple fluid-filled tubules, which causes the dentin to be moist and hydrophilic, presenting a challenge for the interactions of modern adhesives with dentin [63,65]. In simplified E&R systems, the dentin must not be overly dried, since this could cause the collagen fibrillar network to collapse, preventing adequate hybridization and leading to a low bond strength and postoperative sensitivity [73]. SE adhesives seem to create a more homogenous hybrid layer, simultaneously etching and infiltrating the dentin, with a lower level of postoperative sensitivity [74]. If both enamel and dentin are present as the bonding substrates for cementation, it is advisable to selectively etch the enamel prior to bonding [56].

### 2.4. Pretreated Dentin as a Substrate for Adhesive Cementation

After certain dental procedures, such as bleaching, or endodontic treatment (use of endodontic irrigants and root canal sealing materials), adhesive cementation can be negatively affected. Following a bleaching procedure, residual oxygen from the hydrogen peroxide bleaching agent can be trapped on the surface of the tooth [75]. This can prevent the polymerization of resins and consequently adversely affect bond strength [75,76,77]. Hydrogen peroxide can also affect adhesive polymerization when used as an endodontic irrigant, along with sodium hypochlorite, which releases oxygen and water, potentially impairing polymerization [78]. Furthermore, eugenol-containing materials used in endodontics, such as certain endodontic sealants and temporary zinc-oxide–eugenol materials can contaminate dentin and inhibit polymerization [79] because eugenol acts as a radical scavenger [80]. Therefore, the surface of dentin needs to be mechanically and chemically cleaned to remove the agents that can adversely affect polymerization [81,82,83,84], or in the case of bleaching, a restoration should not be made immediately [85,86].

Contrary to this, there have recently been reports on the pretreatment of dentin with certain aqueous or ethanol-based primers containing cross-linkers and MMP inhibitors that enhanced the longevity of the resin–dentin interface. Cross-linkers were shown to inactivate endogenous dentinal enzymes and create chemical bonds between collagen molecules, reinforcing the dentin structure. For instance, carbodiimide-based [67,87,88,89,90,91] and proanthocyanidin-based primers [92,93,94] inactivated MMPs and enhanced the bond strength of dentin for up to 5 years of artificial aging, while chlorhexidine was found to inhibit MMPs and preserve the integrity of the hybrid layer after 10 years of aging in artificial saliva [95]. To facilitate adhesive procedures and reduce the number of clinical steps, efforts have been made to incorporate certain protease inhibitors into adhesive resins. It was demonstrated that chlorhexidine can be blended into dental adhesives without jeopardizing their polymerization quality [96]. A commercially available adhesive resin containing chlorhexidine demonstrated a higher bond strength and better preservation of adhesive strength after 1 year of artificial aging compared with a chlorhexidine-free adhesive [66].

### 2.5. Immediate Dentin Sealing

**Figure 5:**
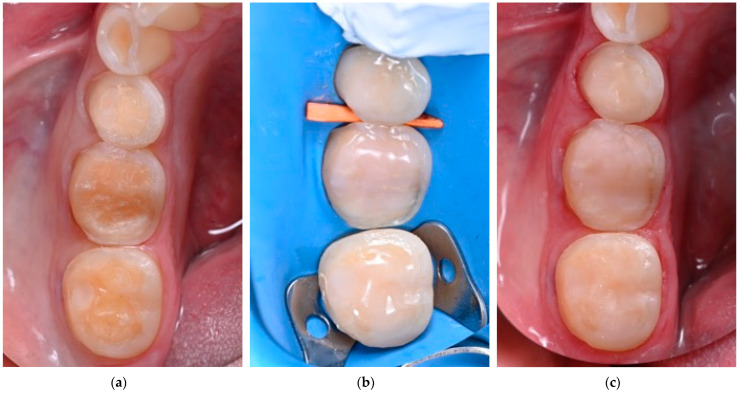
Reinforced immediate dentin sealing on teeth prepared for indirect partial restorations. (**a**) Teeth immediately after the preparation; (**b**) teeth immediately after the application of reinforced IDS under rubber dam isolation; (**c**) teeth after the application of reinforced IDS after the removal of rubber dam.

In an attempt to protect dentin that has been freshly prepared for a partial restoration, obtain lower postoperative sensitivity (POS), and improve bonding and cementation strength, the immediate dentin sealing (IDS) technique was introduced [97,98]. IDS involves applying and polymerizing an adhesive system to freshly exposed dentin immediately after tooth preparation for an indirect restoration. While a three-step EAR adhesive was initially recommended for IDS, it was also demonstrated that two-step self-etch (SE) and universal adhesives are also effective for this purpose [99]. Additionally, a layer of flow resin composite can be applied onto an adhesive (reinforced IDS; Figure 5), providing additional protection and, in cases of partial indirect restorations, the possibility to improve the geometry of the preparation [100]. Although many in vitro studies support the benefits of IDS in terms of bond strength, bacterial microleakage, and POS [101,102], a recent systematic review found no significant effect on POS [103], highlighting the need for more high-quality clinical trials to validate the technique’s efficacy.

### 2.6. Substrate Decontamination Before Cementation

Generally, the fabrication of indirect restorations requires time. Hence, provisional restorations need to be placed on substrates to maintain the integrity of the tissues until the final restoration is fabricated. Provisional crowns are sustained by provisional cements, which can influence the outcome of definitive crown cementation due to several factors. Mainly, the residue contamination alters the surface free energy and reduces bond strength with the substrate. The restorative fit checking medium is also a surface contaminant and can decrease bond strength. Thus, contaminants should be completely removed prior to definitive cementation [104,105,106].

Several methods of surface contaminant removal from bonding substrates have been suggested, such as air-borne particle abrasion, hand cleaning with an excavator, ultrasonic cleaning, chemical agents, pumice with rotary brush cleaning, and a combination of two or more of these methods. However, these methods have shown different results regarding the definitive luting after the procedure. Firstly, hand cleaning with an excavator was reported to not completely remove the remnants of the provisional cement, microscopically appearing in surfaces that macroscopically seemed to be clean [107]. Furthermore, ultrasonic cleaning was reported as more efficient in removing zinc-oxide provisional cement compared with organic solvents. Also, air-bone particle abrasion demonstrated a positive influence on the bond strength of resin cements. Hence, the combination of ultrasonic cleaning and air-bone particle abrasion is reported to present a higher adhesive bond strength in comparison with all other methods [106,108,109].

A recent study evaluated the effect of temporary cement cleaning methods on the retention of crowns cemented using zinc phosphate and resin-modified glass ionomer cement as definitive cements. Different behaviors were observed depending on the cleaning method. For zinc phosphate cement, cleaning with an orange solvent, air-borne particle abrasion, and the ultrasonic method was effective, but the best bond strength results were observed with the air-borne abrasion technique. However, when definitive cementation was performed with resin-modified glass ionomer cement, better results were obtained with the orange solvent or ultrasonic cleaning methods, with no differences between them [107]. Recently, a functional monomer containing a primer originally intended for cleaning zirconia surfaces before cementation (Katana^TM^ Cleaner (KC), Kuraray Noritake Dental Inc., Tokyo, Japan) was tested for efficiency in cleaning dentin surfaces contaminated with saliva or temporary cements before adhesive and cementation procedures. It was demonstrated that this cleaning agent efficiently decontaminated and restored the adhesive properties of dentin [110,111] without negatively influencing its structure and nanomechanical properties [112].

### 2.7. Build-Ups as Substrates for Cementation

**Figure 6:**
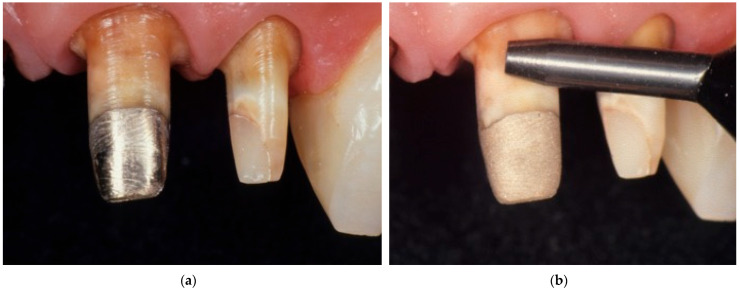
Tooth abutments restored with different restorative materials prepared for complete crowns: (**a**) the abutments after the preparation; (**b**) abutments after air-particle abrasion.

Teeth with an extensive losses of tissue require preparation for indirect restoration. This preparation can include build-ups of different materials, such as fiber posts, zirconium oxide or metal posts in combination with resin composites, compomers, or glass ionomer cements. With advancements in the understanding of the biomechanical properties of the residual tooth structure in severely compromised teeth and the development of appropriate techniques for the management of such a substrate, a wide range of clinical situations can be resolved with composite resin build-ups without opting for more invasive and complex solutions such as surgical crown lengthening or orthodontic extrusion [113,114]. Also, other materials, such as amalgam and metal alloys, can be used as restoration or abutment materials, or these abutment materials can be found underneath old prosthodontic restorations that need to be replaced (Figure 6) [115]. Resin-based cements have been widely used for the cementation of indirect restorations due to their capacity for adhesion to tooth structures and restorations, as well as their clinically acceptable mechanical and esthetic properties [116]. However, there are differences in the bond strength of resin cements to different abutment materials. The highest bond strength values have been reported in materials that are chemically and/or mechanically similar to the bonding system, such as compomers and resin composites. Also, glass ionomer cements present relatively high bond strength values, but these values are lower compared with resin composites [115,116].

On the other hand, the lowest bond strength values and the highest rates of adhesive failures have been observed in cementation with amalgam or gold as abutment materials. It is suggested that these types of substrates should be removed and replaced by restorative options that can provide an acceptable bond strength after cementation. However, these materials can still be maintained if air-particle abrasion is performed, increasing the micromechanical retentive surface for the infiltration of the cement and improving bond strength [115,116]. Micromechanical abrasion can be enhanced with a tribochemical surface modification if air-particle abrasion is performed with particles coated in a thin layer of silica, followed by the application of a silane coupling agent [117].

Regarding adhesive cementation to resin-based substrates, the aging of resin-based abutments can influence bond strength. While freshly polymerized resins have 40–50% unreacted methacrylate groups that allow for the adhesion of new resin layers, aged resin has not demonstrated the same behavior. Unreacted methacrylate groups are reduced over time, thereby also reducing the bond strength. Also, the use of polishing instruments accelerates the reduction of these reactive groups, exposing inorganic filler particles to the surface, which can further reduce bonding capacity. It was shown that the bonding of aged resin-composites could reach 20–80% of the initial bond strength presented by freshly polymerized resins [118,119].

All the abovementioned considerations are summarized in Table 1 with recommendations on the substrate preparation and cementation protocols to employ in different clinical cases.

### 2.8. Pulp Protection and Material Toxicity

Theoretically, dentin hybridization should provide a strong and durable micro-mechanical retention of resins and the complete sealing of restorations without the risk of pulpal toxicity [120]. When such conditions are clinically obtained, the risk of pulpal irritation by adhesive restorations is prevented. It is interesting to note that even in the presence of strong adhesion, incompletely infiltrated hybrid layers can still permit leakage through submicroscopic porosities within the hybrid zone, and most adhesive systems cannot eliminate the passage of fluid across bonded interfaces due to defective hybridization [121].

Despite biocompatibility studies reporting that bacterial leakage is more likely to cause adverse effects to dental pulp than components from restorative materials, other reports have indicated that resin molecules are toxic at micro-molecular concentrations [122,123]. The risk of toxic effects is further increased by the potential of adhesive materials to leach into pulpal tissues given the tubular structure of dentin. During bonding procedures, the risk of chemical irritation is present. Such irritation could undoubtedly trigger an immediate pulpal response. Fortunately, the concentration of leached components from resin composites does not appear to cause acute toxicity to odontoblasts, and most observed reactions are therefore minor and reversible [124].

More interesting are the long-term effects on pulpal cells resulting from the progressive degradation of poorly polymerized adhesive resins diffusing down to the pulp. Studies have indicated that very low concentrations of resin monomers, which are known to pass through dentin by diffusion, can have significant effects on the proliferation and activity of human monocyte–macrophages [125]. The potential for bacterial injury to the dental pulp may be enhanced since its resistance to infectious agents is decreased. When bacterial leakage occurs with the progressive biodegradation of adhesive resins during clinical service, the problem is likely to become more acute.

Moreover, a high toxicity of monomers able to reach the pulp through dentin tubule diffusion, such as 2-hydroxyethyl methacrylate (HEMA), urethane dimethacrylate (UDMA), bisphenol A-glycidyl methacrylate (Bis-GMA), and triethylene glycol dimethacrylate (TEGDMA), was reported [126]. This diffusion is increased when the conditioning procedure was performed prior to the application of resin-based materials, increasing the toxicity of these materials. However, self-adhesive resin cements and resin-modified glass ionomer cements do not require the prior application of conditioning acid, creating a hybrid layer that does not result in the formation of long resin tags in the dentin tubules, which is a safer protocol for cementation in deep cavities that prevents severe toxicity to the pulp tissue [126,127,128]. Recently, however, it was demonstrated that universal resin cements can also influence cell viability, depending on the polymerization mode and with differences between the products [129]. Although it is evident that the monomers of resin cements pose a certain level of toxicity, it is important to mention that this is within the acceptable range and these materials are therefore considered safe to use. It is important to underline that the adequate manipulation of cement and adequate polymerization not only help the obtainment of the highest bond strength to substrates but also reduce the elution of unpolymerized monomers and, consequently, toxicity [99,130].

## 3. Discussion and Future Perspectives

This manuscript presents a review of the literature on the substrate factors that influence cementation, and its clearly demonstrates that substrates play a significant role in the bonding process and the overall success of cementation. Based on the thorough review of the literature presented in this manuscript, it is clear that, unfortunately, it is not possible to provide a single “recipe” for the cementation of indirect restorations that would be adequate for all substrates, but considerations need to be made in each individual case. However, there are certain rules to be applied whenever cementing an indirect restoration. Firstly, whichever the cementation strategy, the substrate needs to be adequately prepared and cleaned from impurities that could interact with the cement or hinder the optimal interaction of the cement with the substrate. In that regard, sandblasting the surface of the substrate can offer important advantages regardless of the abutment material by cleaning and creating a microretentive surface texture. Also, pumice and cleaning primers (such as Katana cleaner) can be recommended. Furthermore, all cementation materials should be used strictly according to the manufacturers’ instructions so as to obtain the material’s optimal physical properties and longevity. This is particularly the case with resin cements, which offer the highest bond strength and stability for indirect restoration but also require the most attention and procedural rigor during the cementation procedure. Each abutment should be considered a separate clinical scenario, and different techniques can be used simultaneously. For instance, if both enamel and dentin are present on the abutment, the practitioner can decide to etch the enamel but use an SE or self-adhesive technique for dentin. If the abutment has a cast metal build-up, a primer/adhesive containing the 10 MDP monomer should be applied, since this monomer chemically binds both to tooth tissues and metal and improves adhesion to these substrates.

Advancements in adhesive dentistry are ever more focused on creating materials that are more biocompatible, have better chemical and physical interactions with cementation substrates, and are more universal—materials that work well on all types of substrates in the hands of expert and inexperienced practitioners [8]. Certain resin cement manufacturers are adding novel monomers that chemically bind to dental tissues and polymerization initiators that are efficient in both hydrophobic and hydrophilic conditions into the composition of their products [10]. In the future, the dental industry will possibly also shift to other types of monomers, such as acrylamides [131,132], that are more resistant to hydrolytic degradation, more biocompatible, have antimicrobial properties, and can even chemically bind to dentin collagen [131,132]. Additionally, bisphenol A (BPA)-containing monomers could be (and already are in some cases) excluded from resin cement formulations. BPA is an estrogen promoter and could cause estrogen receptor activation, posing risks for fertility [133]. Furthermore, bioactive cements that can promote dentin remineralization, inhibit bacterial growth and MMP activity, or have self-healing properties will possibly be developed in the future.

Although the goal of absolute universality is still out of reach, contemporary adhesive resins and resin cements are indeed very versatile. For example, universal cements can be used both with and without adhesive resin on the same abutment. This can be useful in cases when a rubber dam cannot be placed, such as in the case of short abutments for crowns. Given that adhesive resins are sensitive to the presence of water, they should not be placed on the cervical portion of the abutment without a rubber dam. However, adhesive cementation offers a higher bond strength compared with self-adhesive cementation, particularly in short abutments. Hence, Breschi et al. described a cementation technique (Selective Adhesive Luting—SAL) that involves the use of universal resin cement systems and the placement of their dedicated universal adhesive only on the coronal half of the abutment while using the self-adhesive technique on the cervical half of the abutment [134]. Therefore, the cementation of indirect restorations is clearly evolving towards smarter, more durable, and simplified workflows. These advancements aim to meet the challenges posed by modern materials and complex clinical scenarios while maintaining biocompatibility and patient safety.

## 4. Conclusions

Based on the comprehensive information provided in the present review, it is clear that the variability of substrates and their specific interactions with cementation materials are very important factors to be considered prior to the cementation of any indirect restoration so as to obtain a successful and durable adhesion. Abutments should be cleaned properly, regardless of their composition and the choice of cementation material. The sandblasting of substrates is advisable in order to clean the abutment surface, increase the cementation surface, and refresh the build-up materials on the abutment if present. If there is enamel tissue on the abutment, it should be etched. Dentin, on the other hand, is a complex tissue that, if cemented with adhesive resin polymer cements, should be carefully bonded with adhesive resin following the instructions for use and after choosing the optimal modality based on the dentin’s condition and depth. Clinical decisions should be substrate- and case-specific and will undoubtedly influence the longevity of indirect restorations.

## Figures and Tables

**Table 1:** Recommendations for cementation on different substrates.

Substrate	Preparation	Methacrylate-Based Cements (Self-Adhesive Cements, Adhesive Cements That Require a Separate Bonding Agent Application, Universal Cements—Can Be Used with or Without an Adhesive System)	Conventional Cements (Zinc Phosphate, Zinc-Oxide–Eugenol Cement, Zinc Polycarboxylate Cement, GIC, Resin-Modified GIC)
Enamel	Removing aprismatic surface Cleaning with ultrasound and air-borne particle abrasion Etching with 35–37% phosphoric acid for 30 s	Etch-and-rinse adhesives and cements or universal cements used with and adhesive in etch-and-rinse mode are recommended	Not recommended due to low adhesive bond strength
Superficial dentin	Cleaning with ultrasound and air-borne particle abrasion Etching with 35–37% phosphoric acid for 15 s and blot drying or no treatment (depending on the adhesive system)	Etch-and-rinse or self-etch adhesives and cements, self-adhesives, and universal cements are recommended	Caries-affected dentin: glass ionomer cements Other cements are applicable depending on abutment configuration and esthetic factors
Deep dentin	Cleaning with ultrasound and air-borne particle abrasion	Self-etch adhesives and cements, self-adhesives, and universal cements are recommended	Caries-affected dentin: glass ionomer cements Other cements are applicable depending on abutment configuration and esthetic factors
Root dentin	Mechanical cleaning of the root canal Cleaning with EDTA or ultrasound-activated EDTA to remove the debris	Dual-curing resin cements are recommended	Conventional cements are applicable
Enamel and dentin	Cleaning with ultrasound and air-borne particle abrasion Selective etching of the enamel for 30 s	Self-etch adhesive and cements, self-adhesives, and universal cements are recommended	Caries-affected dentin: glass ionomer and resin-modified glass ionomer cements Other cements are applicable depending on abutment configuration and esthetic factors
Build ups	Roughening the surface with a fine bur or Cleaning with ultrasound and air-borne particle abrasion	For resin cores: freshly placed resin cores are recommended For metal cores: self-adhesive or universal cements used in self-adhesive mode are recommended (MDP monomer) For combinations of dentin and metal/resin: self-adhesive cements or universal cements used in the self-adhesive mode are recommended	Different cements are applicable depending on abutment configuration
